# Structures of adenosine receptor A_2B_R bound to endogenous and synthetic agonists

**DOI:** 10.1038/s41421-022-00503-1

**Published:** 2022-12-28

**Authors:** Hongmin Cai, Youwei Xu, Shimeng Guo, Xinheng He, Jun Sun, Xin Li, Changyao Li, Wanchao Yin, Xi Cheng, Hualiang Jiang, H. Eric Xu, Xin Xie, Yi Jiang

**Affiliations:** 1 CAS Key Laboratory of Receptor Research, Shanghai Institute of Materia Medica, Chinese Academy of Sciences, Shanghai, China; 2 CAS Key Laboratory of Receptor Research, National Center for Drug Screening, Shanghai Institute of Materia Medica, Chinese Academy of Sciences, Shanghai, China; 3 University of Chinese Academy of Sciences, Beijing, China; 4 Zhongshan Institute for Drug Discovery, Shanghai Institute of Materia Medica, Chinese Academy of Sciences, Zhongshan, Guangdong China; 5 School of Pharmaceutical Science and Technology, Hangzhou Institute for Advanced Study, University of Chinese Academy of Sciences, Hangzhou, Zhejiang China; 6 School of Life Science and Technology, ShanghaiTech University, Shanghai, China; 7 Lingang Laboratory, Shanghai, China; 8 State Key Laboratory of Drug Research, Shanghai Institute of Materia Medica, Chinese Academy of Sciences, Shanghai, China

**Keywords:** Cryoelectron microscopy, Extracellular signalling molecules

Dear Editor,

Adenosine (ADO), the most abundant natural nucleoside, is ubiquitously distributed in every human tissue and organ and regulates a multitude of physiological and pathological processes. The physiological functions of ADO are mediated by the adenosine receptors (ARs), which are members of class A G protein-coupled receptors (GPCRs). There are four ARs: A_1_R, A_2A_R, A_2B_R, and A_3_R. ADO binds with relatively high affinity (in nanomolar ranges) to A_1_R, A_2A_R, and A_3_R but with relatively low affinity (in micromolar ranges) to A_2B_R^1^. Upon activation by ADO, A_2B_R couples to both G_s_ and G_q_ proteins to transduce downstream signals^2^. A_2B_R is expressed in many types of cells, including immune cells, fibroblasts, smooth muscle cells, and various tumor cells, and participates in regulating inflammation, cell growth, reactive oxygen species production, cardiac functions, etc. The ADO/A_2B_R signaling plays a tissue protective role in acute disease models, such as myocardial ischemia and acute lung injury, etc.^3^. It also correlates to the regulation of muscle and brown adipose tissue and shows both anti-aging and anti-obesity potential^4,5^. BAY 60-6583, a potent and selective A_2B_R agonist, has cardioprotective effects^6^ and increases the secretion of cytokine in the CD133- or HER2-specific CAR-T cells to eliminate tumor cells^7^. These findings make A_2B_R a potential drug target for the treatment of myocardial ischemia, aging, obesity, cancer, etc. Here, we present two cryogenic electron microscopy (cryo-EM) structures of A_2B_R bound to the endogenous ligand ADO, or to the selective agonist BAY 60-6583, and coupled to a modified G_s_ protein (designated as G_s_ in this paper) at 3.2 Å and 2.9 Å, respectively (Fig. 1a–d; Supplementary Figs. S1–S3 and Table S1). The structures provide unique insights into ADO binding by A_2B_R and a basis for the design of subtype-specific ligands for drug discovery targeting the AR system.

**Fig. 1:**
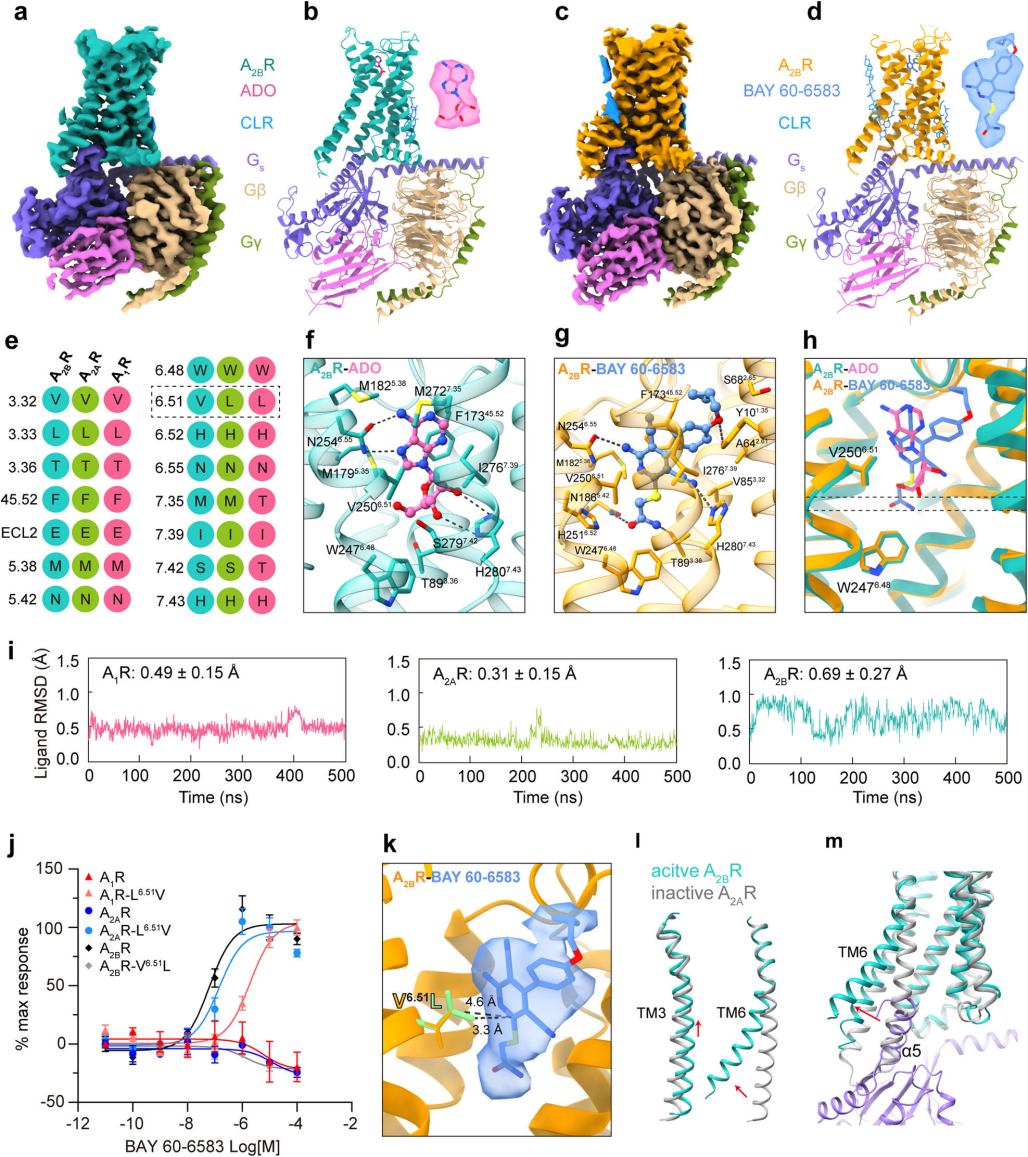
Cryo-EM structures of A_2B_R bound to the endogenous ligand ADO and a selective non-nucleoside agonist BAY 60-6583. **a**, **b** Cryo-EM map (**a**) and structural model (**b**) of the ADO–A_2B_R–G_s_ complex. **c**, **d** Cryo-EM map (**c**) and structural model (**d**) of the BAY 60-6583–A_2B_R–G_s_ protein complex. The ADO (**b**) and BAY 60-6583 (**d**) with their density maps are shown. **e** The sequence alignment of the residues in the ADO-binding pocket among three ARs. **f** ADO-binding pocket in A_2B_R. Hydrogen bonds are shown as black dashed lines. **g** BAY 60-6583-binding pocket in A_2B_R. **h** Structure superposition of ADO– and BAY 60-6583–A_2B_R complexes. Two dashed lines indicate the inserting depth of ADO and BAY 60-6583. **i** The RMSDs of ADO in A_1_R, A_2A_R, and A_2B_R binding pockets. **j** Effects of BAY 60-6583 on the wild-type and mutated ARs with the swapped leucine/valine at position 6.51. NanoBiT Assay was performed to evaluate ligand activity in three independent experiments in triplicate (*n* = 3). **k** Potential steric hindrance between BAY 60-6583 and L^6.51^. The mutation was generated by the software PyMOL. **l**, **m** Conformational comparison of A_2B_R and the inactive A_2A_R (PDB: 4EIY).

In both structures, the receptor and G protein are sufficiently clear for model building (Supplementary Fig. S4). The overall receptor structures comprise canonical seven transmembrane helices (TM1–TM7), three intracellular loops (ICLs), and three extracellular loops (ECLs). Except for the part of ECLs, ICL3, and the C-terminus of the receptor, the rest of the structures are well-defined. Both ADO and BAY 60-6583 are clearly visible within the ligand-binding pocket (Fig. 1b, d). Two A_2B_R complexes exhibit a high similarity with a root mean squared deviation (RMSD) of 0.502 Å. Thus, the well-defined structures can provide invaluable information on ligand–receptor binding and receptor–G protein coupling.

The endogenous agonist ADO binds to the orthosteric binding pocket of A_2B_R in a highly conserved mode across ARs, primarily through hydrogen bonds and hydrophobic interactions (Fig. 1e; Supplementary Figs. S5, S6). A structural comparison of A_2B_R with A_1_R (PDB: 7LD4)^8^ and A_2A_R (PDB: 2YDO)^9^ bound to ADO reveals that adenine moieties, the ADO core, are highly overlapped, while the hydroxyl group in the C5-ribose of ADO shows an orientation diversity (Supplementary Fig. S7). The pyrimidine ring of ADO forms a familial π-stacking with F^45.52^ (superscripts refer to Ballesteros–Weinstein numbering) in A_2B_R to stabilize the adenine group (Fig. 1f). In addition, ADO constitutes two conserved hydrogen bonds with side chains of N^6.55^ and H^7.43^. Residues T89^3.36^, M179^5.35^, M182^5.38^, I276^7.39^, W247^6.48^, M272^7.35^, and S279^7.42^ also contribute to ADO-induced A_2B_R activation (Fig. 1f; Supplementary Table S2). In addition, the intricate water network that exists in A_2A_R is absent in A_2B_R, probably attributed to the limitation of the resolution.

The non-nucleoside agonist BAY 60-6583 adopts a similar binding pose compared with the predicted model in previous molecular docking analysis^10^ (Fig. 1g). Although lacking the core adenine moiety, which is thought critical for ADO binding, it is buried in the identical orthosteric site with a deeper insertion and shows a potent effect on activating A_2B_R (Fig. 1h; Supplementary Fig. S8). The pyridine ring of BAY 60-6583 structurally simulates the pyrimidine group in ADO and makes a similar π-stacking interaction with the side chain of F173^45.52^, while the amine on pyridine of BAY 60-6853 forms a cognate hydrogen bond with N254^6.55^. The acetamide group of BAY 60-6583 forms hydrogen bonds with side chains of T89^3.36^ and N186^5.42^. Two additional hydrogen bonds between BAY 60-6853 and Y10^1.35^ and H280^7.43^ are also observed. The majority of residues in the BAY 60-6538-binding pocket contribute to the BAY 60-6583 activity (Fig. 1g; Supplementary Table S2).

ADO shows low affinity and low potency on A_2B_R compared with other ARs^1^. The differences in receptor sequences and the agonist recognition mode provide clues for understanding the agonist selectivity by ARs. ECLs of ARs show a low sequence identity compared with receptor TMD (Supplementary Fig. S5). However, this sequence non-conservation of ECLs does not translate into agonist specificity, as chimeric A_2B_R-ECL_A2AR_, with all three ECLs from A_2B_R replaced by those from A_2A_R, does not affect the activity of ADO and BAY 60-6583 (Supplementary Fig. S9 and Table S2). Residues in the binding pocket across ARs demonstrate high sequence identity except for residues at positions 6.51, 7.35, and 7.42 (Fig. 1e), of which only the residue at 6.51 shows strong consistency with ADO activity. A_2B_R bears a valine at 6.51 versus leucines in A_1_R and A_2A_R (Fig. 1e) and exhibits the weakest response to ADO. However, the low selectivity of ADO for A_2B_R is irrelevant to V/L^6.51^, as swapping V250^6.51^ in A_2B_R for cognate leucine in A_1_R/A_2A_R does not impact ADO activity (Supplementary Table S2). We further explore the ligand RMSD by 500 ns × 3 molecular dynamics simulations to evaluate the binding stability of ADO in ARs. From the highly similar ligand pose, the binding with A_2A_R is the most stable (RMSD = 0.31 Å) over A_1_R (0.49 Å) and A_2B_R (0.69 Å) (Fig. 1i). Hence, ADO in A_2B_R is relatively instable in the binding site and tends to drift out of it, which may explain its weaker binding affinity^11^.

BAY 60-6583 demonstrates high selectivity for A_2B_R over A_1_R and A_2A_R (Fig. 1j; Supplementary Tables S2, S3). The role of the residue at position 6.51 in the selectivity of BAY 60-6583 across ARs was further explored. Substituting V250^6.51^ in A_2B_R by cognate leucine in A_1_R/A_2A_R caused a notable decrease of BAY 60-6583 activity (Fig. 1j). Vice versa, swapping L^6.51^ in A_1_R/A_2A_R with valine remarkably enhanced BAY 60-6583 activity (Fig. 1j; Supplementary Tables S3). These results support the hypothesis that V/L^6.51^ correlates to the BAY 60-6583 selectivity for A_2B_R over A_1_R and A_2A_R, which may be attributed to the potential steric hindrance from the bulkier side chain of leucine (Fig. 1k). This finding provides a basis for designing high-affinity/potency ligands targeting A_2B_R.

Structural comparison of the G_s_-coupled A_2B_R bound to ADO and BAY 60-6583 with the antagonist ZM241385-bound A_2A_R (PDB: 4EIY) reveals that our two A_2B_R structures are indeed in the active state (Fig. 1l; Supplementary Fig. S10). The cytoplasmic ends of TM6 in ADO/BAY 60-6583–A_2B_R complexes show a pronounced outward displacement compared with that in inactive A_2A_R, the hallmark of class A GPCR activation. TM5 undergoes a concomitant outward movement, while TM7 displays an inward shift upon A_2B_R activation (Supplementary Fig. S10a–d). At the bottom of the binding site, ADO and BAY 60-6583 contact with the “toggle switch” W^6.48^ and induce its downward movement and the subsequent swing of F243^6.44^ and the entire TM6 (Fig. 1l; Supplementary Fig. S10e, f). The binding of distinct agonists leads to a half-helical upward movement of TM3 (Fig. 1l) and conserved active-like conformation changes of residues in PIF, DRY, and NPxxY motifs^12^ (Supplementary Fig. S10e, f). The agonism signal propagates downward, eventually leading to the notable movement of helical cytoplasmic ends of receptor helices to accommodate the G protein (Fig. 1m).

In conclusion, we solved two cryo-EM structures of G_s_-coupled A_2B_R bound to its endogenous ligand ADO and a non-nucleoside selective agonist BAY 60-6583. These structures reveal the highly conserved ADO-binding mode across ARs and provide a potential explanation for the low affinity of ADO for A_2B_R. Compared with ADO, BAY 60-6583, an A_2B_R-selective agonist, engages the identical orthosteric binding pocket of A_2B_R but shows a ligand-specific recognition mode. The deeper insertion of BAY 60-6583 resulting in additional hydrophilic interactions with A_2B_R pocket residues and the valine at position 6.51, may contribute to the high selectivity of BAY 60-6583 for A_2B_R. In addition, the agonism signals reflect familial conformation changes upon activation, such as the half-helical upward movement of TM3. Together, our findings provide the basis for understanding the ADO and non-nucleoside ligand recognition of A_2B_R and receptor activation, thereby providing a structural template for drug design targeting A_2B_R. Our structures also add to the pool of knowledge on ligand recognition and activation regulation of ARs.

## Supplementary information

Supplementary information

## Data Availability

The coordinates and cryo-EM density maps have been deposited in the Protein Data Bank and EMDB with accession codes 8HDO and EMD-34676 for the BAY 60-6583–A_2B_R–G_s_ complex and 8HDP and EMD-34677 for the ADO–A_2B_R–G_s_ complex.
