# Supplementary material for: Structures of adenosine receptor A2BR bound to endogenous and synthetic agonists
Source: Cell Discov. 2022 Dec 28;8:140. doi: 10.1038/s41421-022-00503-1 (PMC9794776; doi:10.1038/s41421-022-00503-1)
Supplement: Supplementary file 1 — Supplementary information [file 41421_2022_503_MOESM1_ESM.pdf]

Supplementary information

Structures of adenosine receptor $A_{2B}R$ bound to endogenous and synthetic agonists

Authors: Hongmin Cai[1,*], Youwei Xu[1,*], Shimeng Guo[2,*], Xinheng He[1,3], Jun Sun[2,3], Xin Li[2,3], Changyao Li[1,3], Wanchao Yin[1,3,4], Xi Cheng[1,3,5], Hualiang Jiang[1,3,5,6,7], H. Eric Xu[1,3,6,8,†], Xin Xie[2,3,5,6,8,†], Yi Jiang[6,7,†]

Correspondences: yjiang@lglab.ac.cn (Y.J.); xxie@simm.ac.cn (X.X.); eric.xu@simm.ac.cn (H.E.X.)

This file includes:
Methods
Figures S1 to S10
Table S1 to S3
Supplementary References

Methods

Construct cloning

The gene encodes human $A_{2B}R$ (residues 2-332) was synthesized (Synbio) and subcloned into pFastBac vector containing an N-terminal hemagglutinin signal peptide (HA) and thermostabilized apocytochrome b562RIL (BRIL) using homologous recombination (Clone express one step cloning kit, Vazyme). To improve the stability of the complex, the NanoBiT tethering strategy was used by fusing a LgBiT subunit at the C-terminus of the receptor[1]. To facilitate expression and purification of the complex, two maltose-binding proteins (MBPs) were fused at the C-terminus of the receptor. A tobacco etch virus protease site (TEV site) was inserted between LgBiT and MBP for protease cleavage.

G_s, with a removed α-helical domain, was modified based on the sequence of human G_s with two dominant-negative mutations (G226A and A366S)，which has been used in structure studies of G_s-coupled dopamine receptor 1 complexes[2]. Rat Gβ1 was cloned with a C-terminal HiBiT for structural complementation of LgBiT to form a NanoBiT. The G protein components, G_s, rat Gβ1-HiBiT, and bovine Gγ2, were cloned into a pFastBac vector, respectively.

Insect cell expression

The BRIL-A_{2B}R-2MBP-LgBiT, G_s, Gβ1-HiBiT, and Gγ2 were co-expressed in *Spodoptera frugiperda* (*Sf9*) insect cells using Bac-to-Bac baculovirus expression protocol. Cell cultures were adjusted in serum-free ESF921 medium to a density of 3.5×10^6 cells per mililiter and then infected with baculovirus of BRIL-A_{2B}R-LgBiT-MBP-MBP, G_s, Gβ1-HiBiT, Gγ2 at the ratio of 1:1:1:1. After incubation for 48 h at 27 °C, cells were harvested and stored at -80 °C until use.

Expression and purification of Nb35

Nanobody35 (Nb35) was expressed in *E. coli* BL21(DE3)[3]. Its sequence contained a N-terminal *pel*B signal peptide for secretion into periplasm and a C-terminal 8×His-tag for purification by the Nickel resin (Smart-Lifesciences). Cells were grown in TB medium supplemented with 100 mg/L ampicillin to OD_{600} of 0.8 at 37 °C, further induced with 200 mg/L IPTG for 17 h at 18 °C. Cells were

subsequently lysed by osmotic shock. Cells from 1 L culture were resuspended in 20 mL TES (0.5 M sucrose, 0.2 M Tris-HCl pH 8.0, 0.5 mM EDTA) with stirring at 4 °C for 30 min. After dehydration, cells were rehydrated with 40 mL of ice-cold H_2O at 4 °C for 1 h. The periplasmic extraction was collected by centrifugation at 20,000×g at 4 °C for 30 min. The supernatant was brought to a concentration of 150 mM NaCl, 2 mM $MgCl_2$, and 20 mM imidazole. Equilibrated Nickel resin was incubated with supernatant and washed with 30 mM imidazole in buffer A (150 mM NaCl, 20 mM HEPES pH 7.5). Nb35 was eluted with 300 mM imidazole in buffer A. The eluted protein was further purified by HiLoad 16/600 Superdex 75 column with a size buffer of 100 mM NaCl, 20 mM HEPES pH 7.5. The eluted fractions containing Nb35 were collected and concentrated to 2 mg/mL, and then flash-frozen in liquid nitrogen before storage at -80 °C.

Complex purification

For the purification of both the ADO-A_{2B}R-G_s and BAY 60-6583-A_{2B}R-G_s complexes, cell pellets were thawed at room temperature and resuspended in buffer B (150 mM NaCl, 10%(v/v) glycerol, 20 mM HEPES pH 7.5) supplemented with 5 mM $MgCl_2$, 5 mM $CaCl_2$, 0.2 mM TCEP (Tris-(2-carboxyethyl)phosphine, Hampton Research) and protease inhibitor cocktail (TargetMol). The complexes were formed in the presence of 1 mM adenosine (Sigma) or 10 μM BAY 60-6583 (HY-103171, MedChemExpress), 25 mU/mL apyrase, and 10 μg/mL Nb35, followed by incubation at room temperature with rotating. After incubation, the lysate was solubilized by addition of 0.5 %(w/v) lauryl maltose neopentylglycol (LMNG, Anatrace) and 0.1%(w/v) cholesteryl hemisucinate (CHS, Anatrace) for 2 h at 4 °C. The supernatant was isolated by centrifugation at 65,000×g for 45 min and then incubated with dextrin beads (Smart-Lifesciences) for 3 h at 4 °C. The resin was packed on a gravity column and washed with 20 column volumes of wash buffer contain (100 mM NaCl, 2 mM $MgCl_2$, 0.2 mM TCEP, 1 mM adenosine or 5 μM BAY 60-6583, 0.01 %(w/v) LMNG, 0.002 %(w/v) CHS, 20 mM HEPES pH 7.5). The protein was eluted with wash buffer containing 10 mM maltose and treated with TEV protease overnight at 4 °C. Complex and TEV protease were concentrated using a 100 kDa molecular weight cut-off Vivaspin Ultrafiltration concentrator and loaded onto Superdex 200 Increase 10/300 GL column (GE Healthcare) with size-exclusion buffer (100 mM NaCl, 0.1 mM TCEP, 1 mM

adenosine or 5 μM BAY 60-6583, 0.00075 %(w/v) LMNG, 0.00025 %(w/v) glyco-diosgenin (GDN, Anatrace), 0.0002 %(w/v) CHS, 20 mM HEPES pH 7.5). The fractions for the complex were pooled and concentrated to the desired concentration for the cryo-EM study.

Cryo-EM data collection

Cryo-EM grids were prepared with the Vitrobot Mark IV plunger (FEI) set to 4 °C and 100% humidity. Three-microliter of 3 mg/mL purified ADO-A_{2B}R-G_s complex was applied to the glow discharged holey carbon grids (Zhenjiang Lehua Technology Co., Ltd). The sample was incubated for 5 s on the grids before blotting for 3 s (double-sided, blot force 1) and flash-frozen in liquid ethane immediately. The same condition was used for the sample BAY 60-6583-A_{2B}R-G_s complex.

For the ADO-A_{2B}R-G_s dataset, 5,069 movies were collected on a Titan Krios equipped with a Gatan K3 direct electron detection device at 300 kV with a magnification of 81,000, corresponding to a pixel size of 1.04 Å. Image acquisition was performed with EPU Software (FEI Eindhoven, Netherlands). We collected a total of 36 frames accumulating to a total dose of 50 e $Å^{-2}$ s^{-1} over 2.5 s exposure.

For the BAY 60-6583-A_{2B}R-G_s complex dataset, 5,202 movies were collected on a Titan Krios equipped with a Falcon 4 direct electron detection device at 300 kV with a magnification of 96,000, corresponding to a pixel size 0.8 Å. Image acquisition was performed with EPU Software (FEI Eindhoven, Netherlands). We collected a total dose of 50 e $Å^{-2}$ s^{-1} over 2.5 s exposure on each EER format movie[4]. Each movie was divided into 36 frames during motion correction.

Cryo-EM image processing

MotionCor2 was used to perform the frame-based motion-correction algorithm to generate drift-corrected micrographs for further processing. CTFFIND4 provided the estimation of the contrast transfer function (CTF) parameters[5,6].

For the ADO-A_{2B}R-G_s complex dataset, approximately 2,000 particles were manually picked. 2D classes were calculated and used as references for automatic picking. The subsequent steps of particle picking, and extraction were performed

with Relion3.0[7]. A total of 4,199,012 particles were extracted from the cryo-EM micrographs and imported into cryoSPARC[8]. Two rounds of reference-free two-dimensional (2D) classification were used to separate out 1,077,477 particles. The followed two rounds of three-dimensional (3D) classification yielded 194,666 particles after clearance. We then continued the processing in Relion3.0 and refined 194,666 particles, which led to a structure at 3.65 Å global resolution. After CTF refinement, Bayesian polishing, and postprocessing, the particles were reconstituted to a 3.20 Å structure.

For the BAY 60-6583-A_{2B}R-G_s complex dataset, approximately 2,000 particles were manually picked. 2D classes were calculated and used as references for automatic picking. All subsequent steps of particle picking, extraction, classification and post-processing of refined models were performed with Relion3.0[7]. A total of 2,300,698 particles were extracted from the cryo-EM micrographs and followed by reference-free two-dimensional (2D) classification, yielding 1,259,854 particles after clearance. Two rounds of three-dimensional (3D) classification were used to separate out 840,466 particles that resulted in a clearer density of the BAY 60-6583-A_{2B}R-G_s complex. We refined the remained particles, which led to a structure at 3.76 Å global resolution. After CTF refinement, Bayesian polishing, and post-processing, the particles were reconstituted to a 2.87 Å structure.

Model building

A_{2B}R structure predicted from AlphaFold2 was used as the starting reference model for receptor building[9]. Structures of G_s, Gβ1, Gγ2, and the Nb35 were derived from PDB entry 7JVQ[2] and were rigid-body fit into the density. All models were fitted into the EM density map using UCSF Chimera[10], followed by iterative rounds of manual adjustment and automated rebuilding in COOT[11] and PHENIX[12], respectively. The model was finalized by rebuilding in ISOLDE[13], followed by refinement in PHENIX with torsion-angle restraints to the input model. The final model statistics were validated using Comprehensive validation (cryo-EM) in PHENIX[12] and provided in the supplementary information, Table S1. All structural figures were prepared using Chimera[10], Chimera X[14], and PyMOL (Schrödinger, LLC.).

cAMP accumulation assay

The wild-type $A_{2B}R$ was subcloned in the pcDNA3.0 vector with an N-terminal HA signal peptide. Mutations were introduced by QuickChange PCR. All the constructs were verified by DNA sequencing. HEK293 cells were cultured in 1 × DMEM supplemented with 10% (v/v) fetal bovine serum and incubate in 5% CO_2 at 37 °C. For transient transfection, approximately 2 × 10^6 cells were mixed with 1 μg of plasmids in 200 μL of transfection buffer, and electroporation was performed with a Scientz-2C electroporation apparatus (Scientz Biotech, Ningbo, China). After 24 h culture, Cells were harvested and re-suspended in PBS containing 500 μM Isobutylmethylxanthine at a density of 2 × 10^5 cells/mL. Cells were then plated onto 384-well assay plates at 1000 cells/5 μL/well. Another 5 μL buffer containing compound at various concentrations were added to the cells and the incubation lasted for 30 min at 37 °C. Intracellular cAMP levels were tested by a LANCE Ultra cAMP kit (PerkinElmer, TRF0264) and EnVision multiplate reader according to the manufacturer's instructions.

NanoBiT assay

To monitor the interaction between G proteins and A_1R, $A_{2A}R$ or $A_{2B}R$ upon agonist stimulation, a NanoLuc-based enzyme complementation system called NanoBiT assay[15] was used (Promega). The C terminus of ARs was fused with the small fragment (SmBiT), and the large fragment (LgBiT) element was fused to the N terminus of $G\alpha_s$ or $G\alpha_i$ proteins. HEK293 cells seeded at 4 × 10^5 cells/well on 96-well plates were co-transfected with plasmids encoding AR-SmBiT with LgBiT-Gα protein by the ratio of 1:1. Twenty-four hours later, cells were replaced with 40 μL of fresh culture medium (without FBS). And then added 10 μL Nano-Glo Live Cell reagent according to the manufacturer's protocol (Promega, Cat#N2011), incubated in a 37 °C, 5 % CO_2 incubator for 5 min. Another 25 μL culture medium with various concentrations of compounds were added to the cells. After incubation at room temperature for 10 minutes, bioluminescence was measured with an EnVision multiplate reader (PerkinElmer).

Cell-surface expression assay

Cell-surface expression for each mutant was monitored by a fluorescence-activated cell sorting (FACS) assay. In brief, the expressed cells were incubated

with mouse anti-HA-FITC antibody (Sigma, H7411) for 20 min at 4 °C, and then a 9-fold excess of PBS was added to cells. Finally, the surface expression of $A_{2B}R$ was monitored by detecting the fluorescent intensity of FITC using a BD ACCURI C6.

Statistical analysis

All functional study data were analyzed using Prism 8 (GraphPad) and presented as means ± S.E.M. from at least three independent experiments. Concentration-response curves were evaluated with a three-parameter logistic equation. EC_{50} is calculated with the Sigmoid three-parameter equation. The significance was determined with one-way ANOVA with Tukey's test, and $^*P < 0.05$ *vs.* wild-type (WT) was considered statistically significant.

Molecular dynamics simulations

The complex of A_1R-adenosine (PDB ID: 7LD4)[16], $A_{2A}R$-adenosine (PDB ID: 2YDO)[17], and $A_{2B}R$-adenosine were used for the construction of MD simulation systems. The missing loops were added back according to loop builder program in Molecular Operation Environment, while other missing structures were built according to the AlphaFold2 models[18]. All mutations were changed back referring to the wild-type sequence and the G proteins were removed. Using CHARMM-GUI, the models were inserted in POPC (palmitoyl-2-oleoyl-sn-glycero-3-phosphocholine) lipids with TIP3P waters and 0.15 M NaCl under a periodic boundary condition[19-21]. The force fields for GPCRs, lipids, and adenosine are FF19SB, lipid17, and GAFF2, respectively[20,21]. We then performed the independent 500 ns × 3 MD simulations on Amber 20. During simulations, Langevin thermostat and Berendsen barostat were used for temperature (300 K) and pressure (1 atm) control. Long-range electrostatic interactions were dealed by the Particle mesh Ewald and a 10 Å cutoff was employed for short-range interactions. The SHAKE algorithm was applied to restrain the bond with hydrogens. The RMSD calculation was accomplished by CPPTRAJ[22].

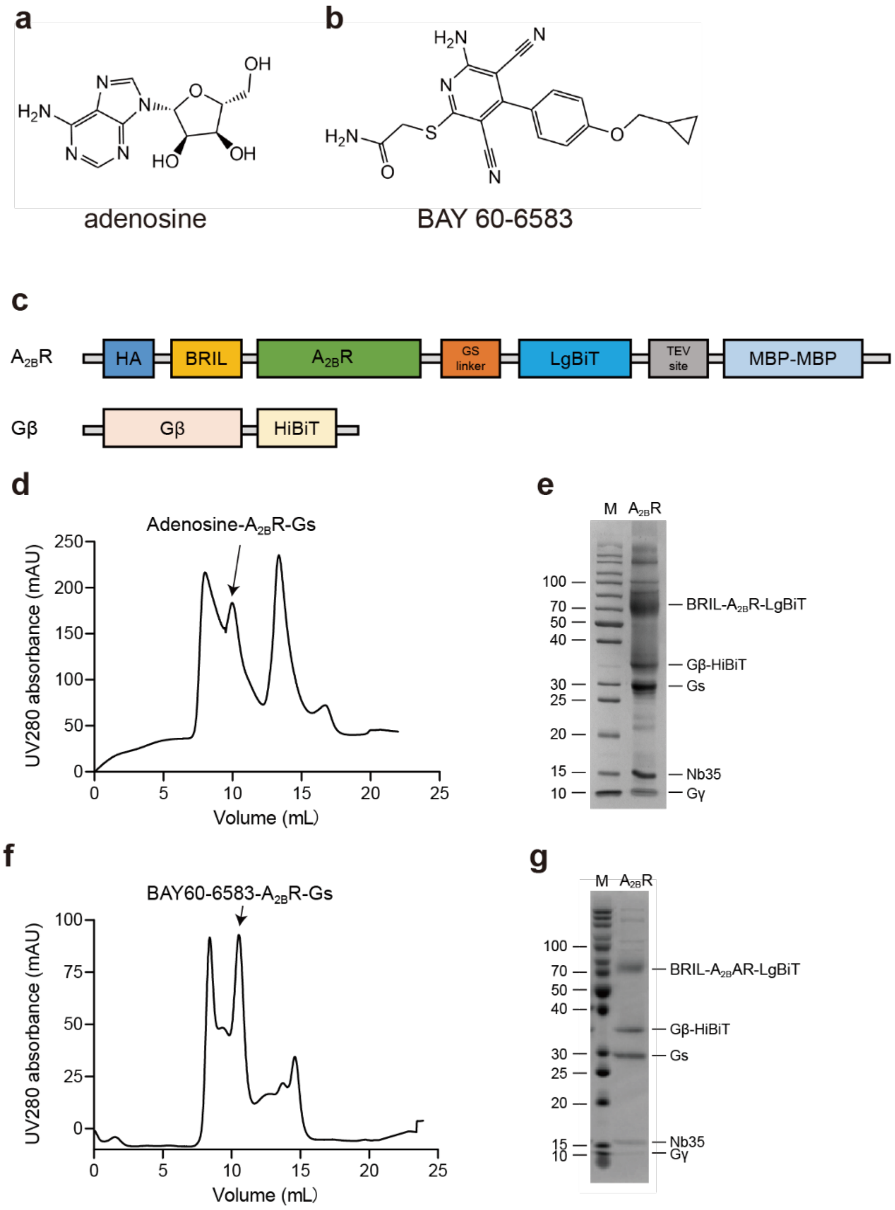

Fig S1. Ligands of $A_{2B}R$ and $A_{2B}R$-G_s complex expression and purification. **a, b,** Chemical structure of adenosine (**a**) and BAY 60-6583 (**b**). **c**, Schematic diagrams of the expression constructs of $A_{2B}R$ and Gβ1 in the structural study. **d,** Size-exclusion chromatography profile of the ADO-$A_{2B}R$-G_s complex. **e**, SDS-PAGE of the arrow indicated peak fraction in (**d**). **f**, Size-exclusion chromatography profile of the BAY 60-6583-$A_{2B}R$-G_s complex. **g**, SDS-PAGE of the arrow indicated peak fraction in (**f**).

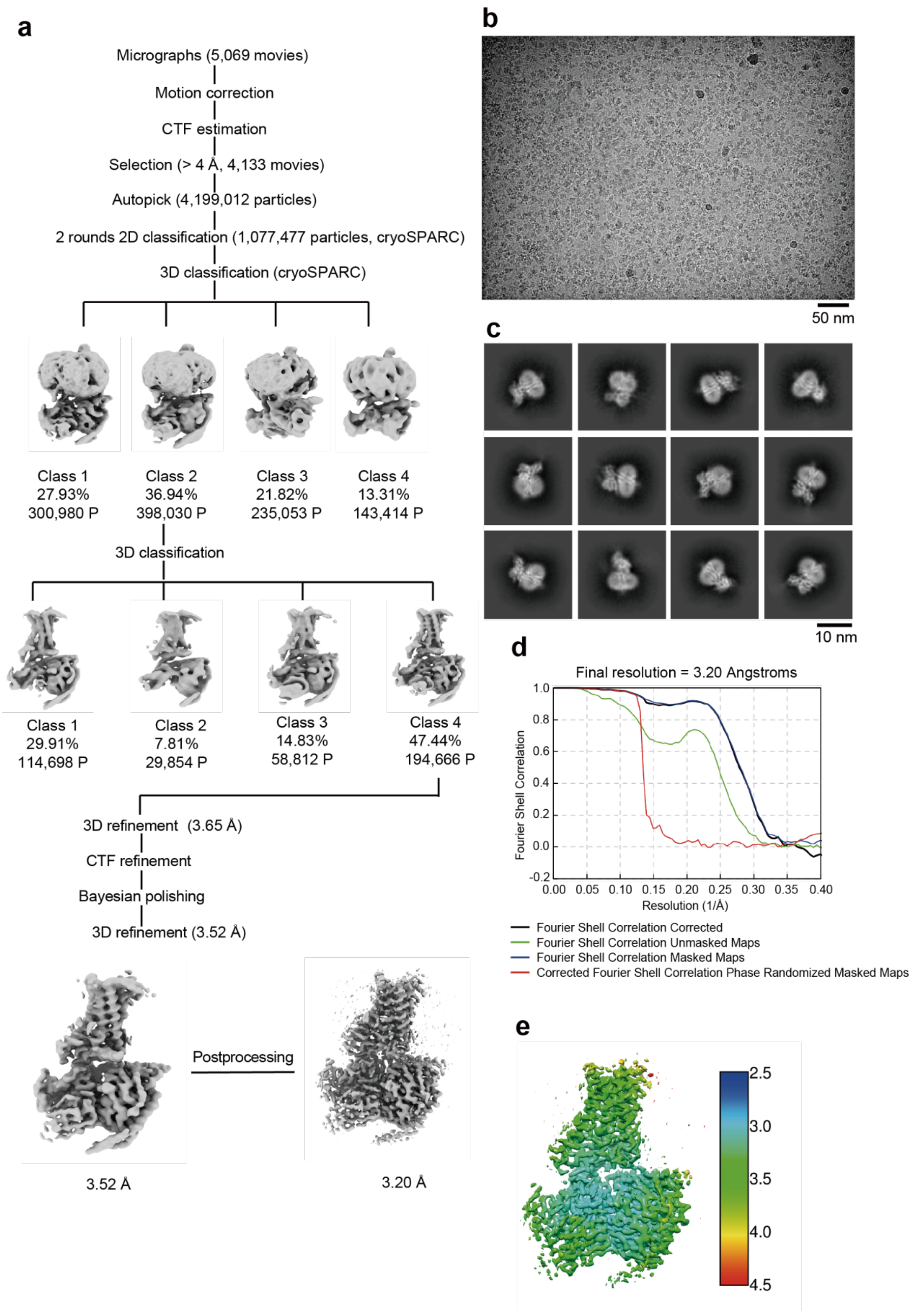

Fig S2. Cryo-EM data processing of the ADO-A$_{2B}$R-G$_s$ complex. a, Flow-chart of the cryo-EM data. **b**, Representative image from cryo-EM. **c**, Representative 2D average classes. Scale bar, 5 nm. **d**, FSC curves. **e**, The local resolution map.

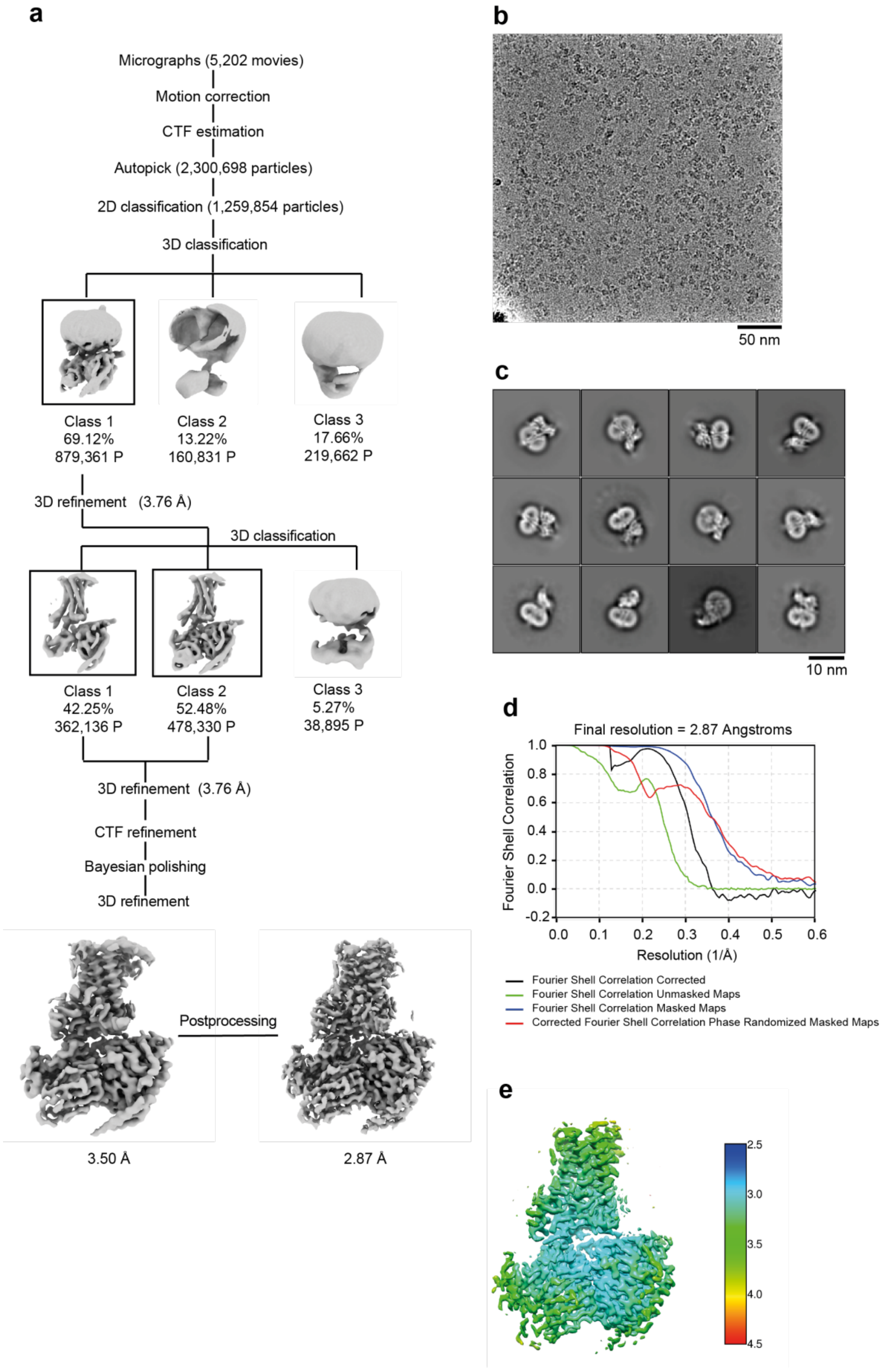

Fig S3. Cryo-EM data processing of the BAY 60-6583-A$_{2B}$R-G$_s$ complex. a, Flow-chart of the cryo-EM data. **b**, Representative image from cryo-EM. **c**, Representative 2D average classes. Scale bar, 10 nm. **d**, FSC curves. **e**, The local resolution map.

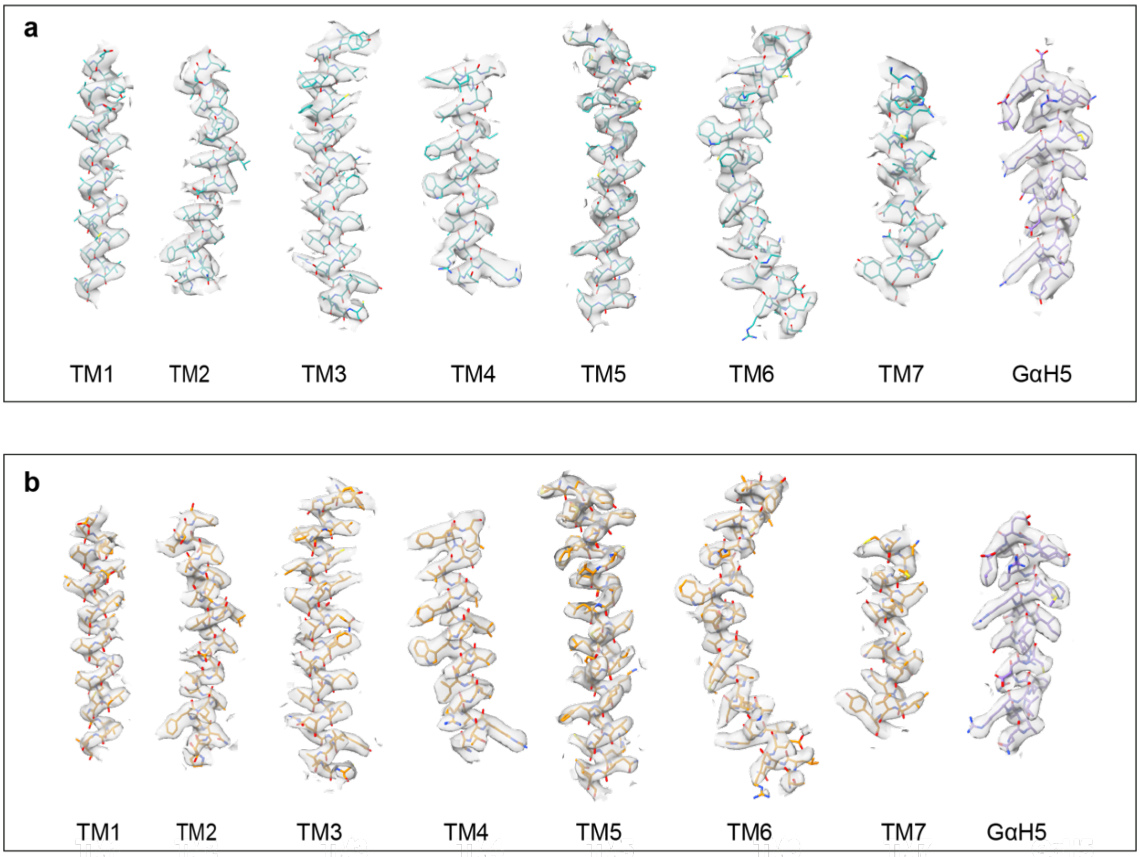

Fig S4. Representative cryo-EM density maps of the ADO-A_{2B}R-G_s and BAY 60-6583-A_{2B}R-G_s complexes. a, b, Cryo-EM density maps of the seven transmembrane domains of ADO- and BAY 60-6583-bound A_{2B}R and the α5 helix of G_s.

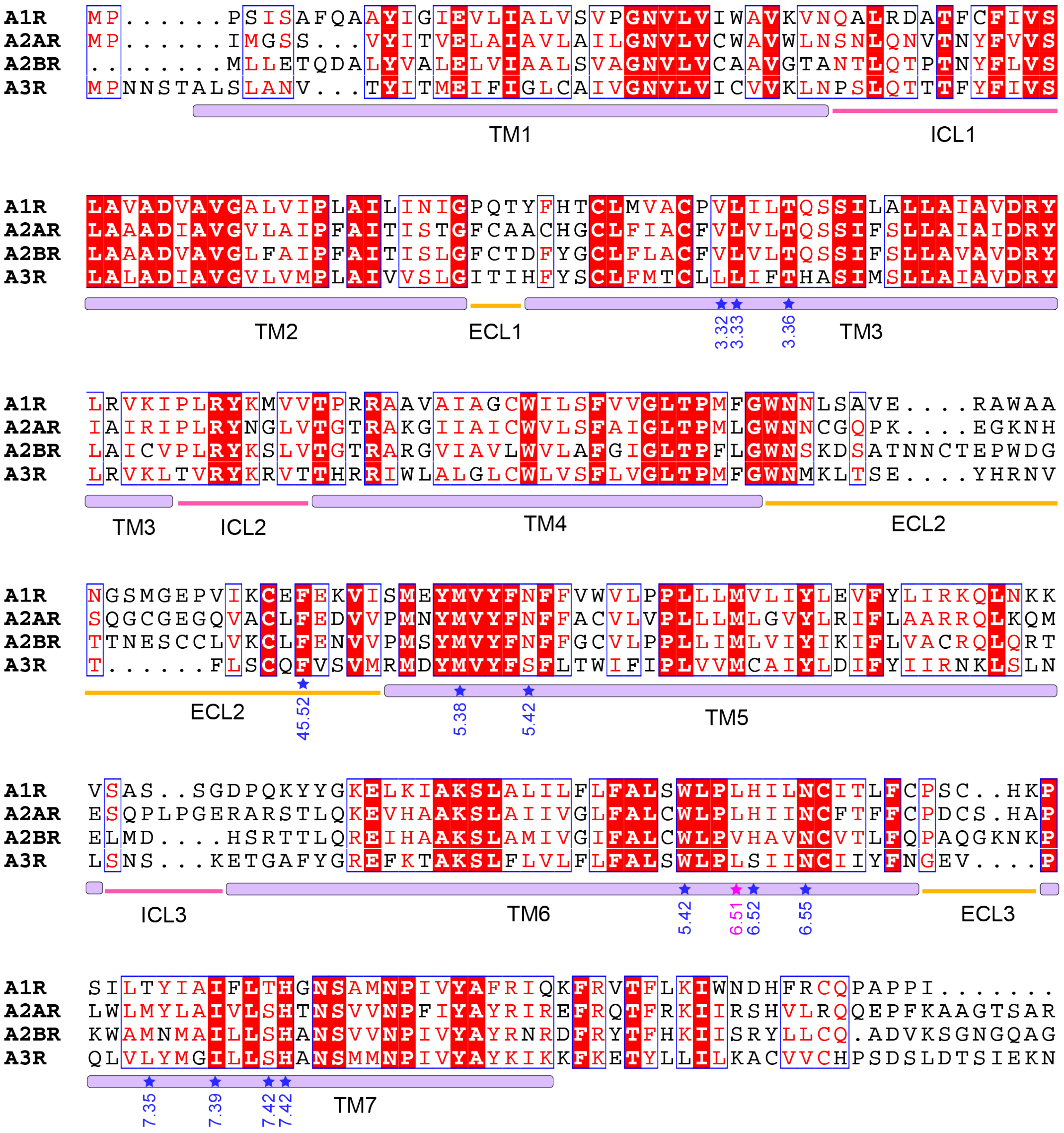

Fig S5. Sequence alignment of adenosine receptors. The graphic was generated on ESPript 3.0 server (https://espript.ibcp.fr/ESPript/ESPript/). Transmembrane domains (TMs), extracellular domains (ECLs), and intracellular domains (ICLs) are labeled. Conserved and similar residues across the family are highlighted at the red background and boxed in blue. The residues in the ADO-binding pocket are labeled with a star and Ballesteros and Weinstein number.

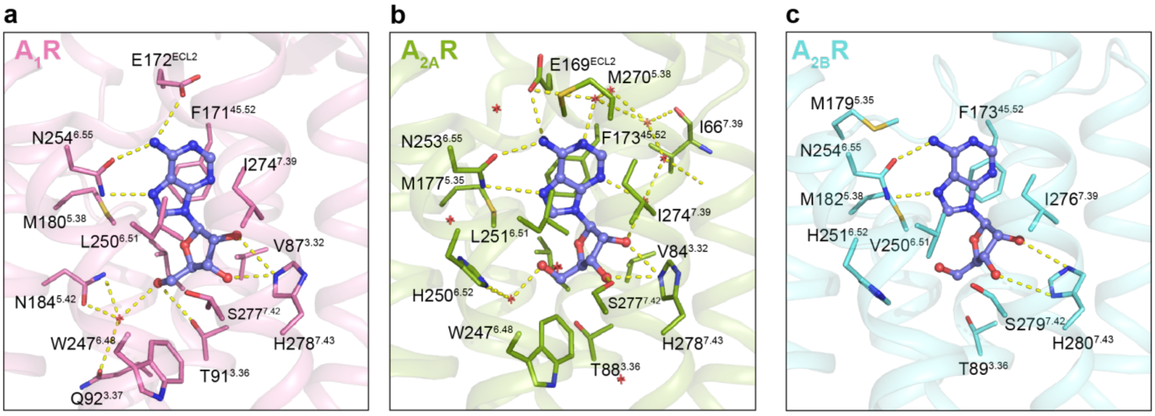

Fig S6. Interactions between ADO and adenosine receptors. **a**, A_1R (PDB:7LD4); **b**, $A_{2A}R$ (PDB: 2YDO); **c**, $A_{2B}R$ in this study. ADO is shown at ball-stick mode and colored in purple. Water molecule is displayed as red stars. Polar interactions are labeled with yellow dash lines.

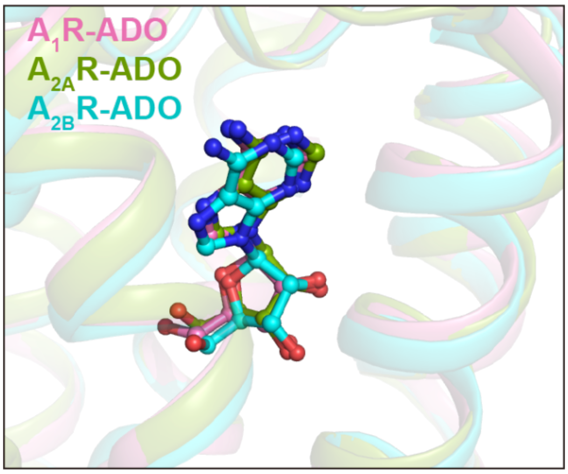

Fig S7. The superimposition of $A_{2B}R$ with A_1R and $A_{2A}R$ to show the adenosine in the orthosteric binding pocket at the similar pose. The adenosine (stick) and corresponding receptor (cartoon) were colored as indicated.

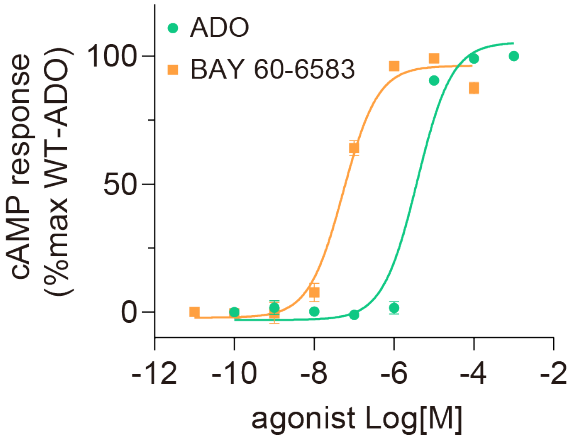

Fig S8. Effect of ADO- and BAY 60-6583-induced cAMP accumulation on $A_{2B}R$.
cAMP accumulation assay was performed to assess endogenous (ADO) and selective compound (BAY 60-6583) activities in a representative experiment.

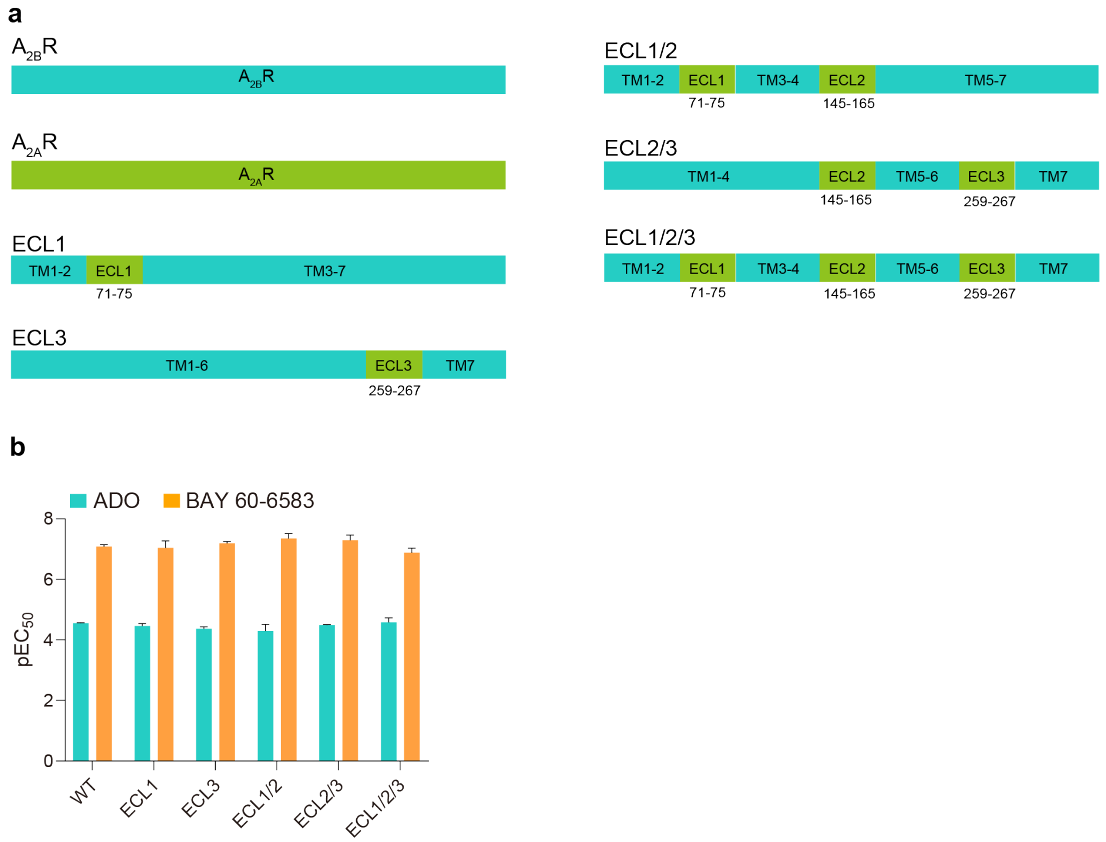

Fig S9. Effects of ECLs on $A_{2B}R$ activation. a, Schematic diagrams of chimera mutant design. The numbering for residues below the constructs were based on the sequence of $A_{2A}R$. **b**, Effects of ECL swapping on the activation of chimeric $A_{2B}R$. NanoBiT assay was carried out to evaluate ADO- and BAY 60-6583-induced chimeric $A_{2B}R$ activation in three independent experiments in triplicate (n = 3).

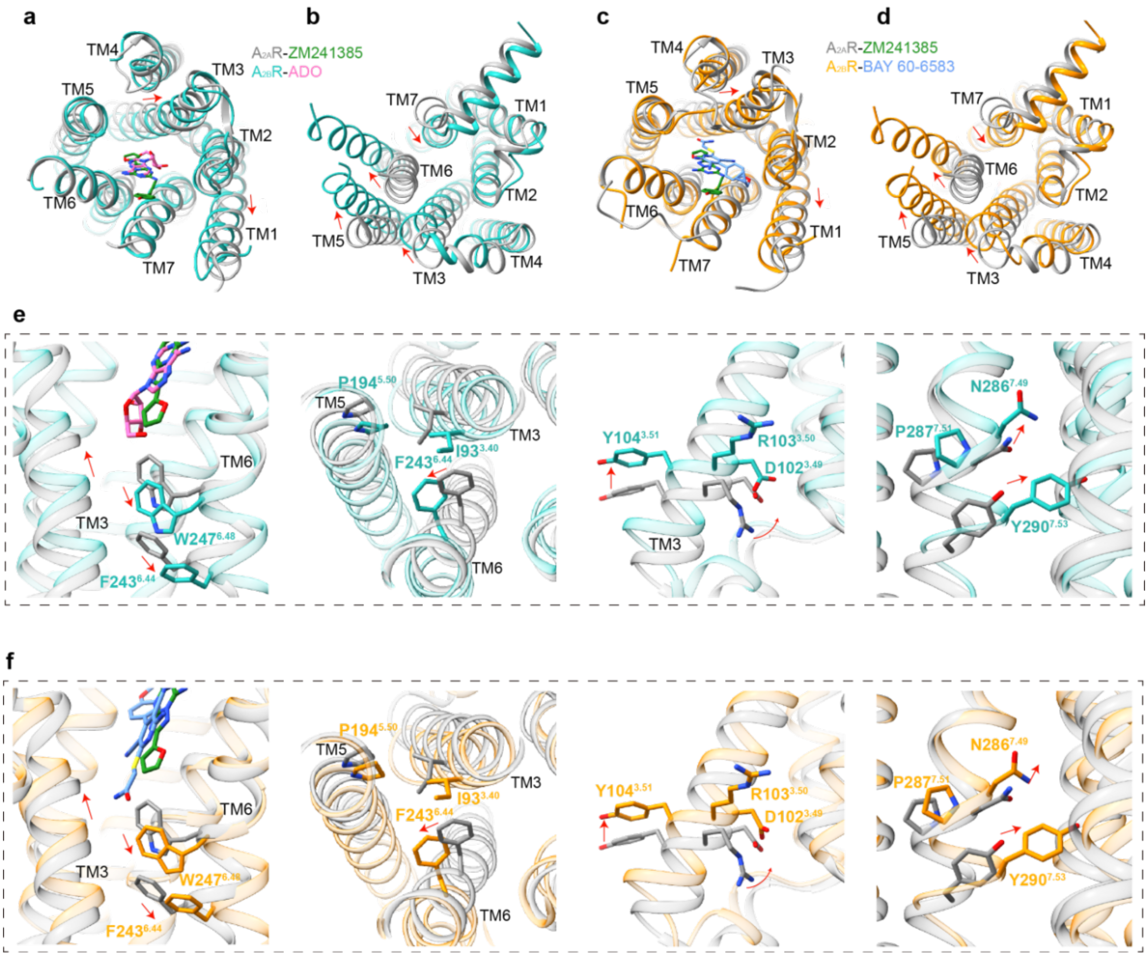

Fig S10. $A_{2B}R$ activation. a, **b**, Structural comparison of the active $A_{2B}R$ (cyan) bound to ADO (pink) and inactive $A_{2A}R$ (grey) bound to antagonist ZM241385 (green) at the extracellular view (**a**) and cytoplasmic view (**b**). **c**, **d**, structural comparison of the active $A_{2B}R$ (orange) bound to BAY 60-6583 (blue) and inactive $A_{2A}R$ (grey) in complex with ZM241385 (green) at the extracellular view (**c**) and the cytoplasmic view (**d**). **e**, **f**, Conformation changes of the conserved motifs, including toggle switch, PIF, DRY, and NPxxY motifs in ADO- (**c**) and BAY 60-6583-bound $A_{2B}R$ (**d**) relative to ZM241385-bound $A_{2A}R$. Movement orientations of the residues in micro switches of the active $A_{2B}R$ relative to the inactive $A_{2A}R$ are indicated by red arrows. The ligand and receptor are colored as indicated in **a-d**.

Table S1. Cryo-EM data collection, model refinement and validation statistics.

	BAY-A_{2B}R-G_s complex	ADO-A_{2B}R-G_s complex
**Data collection and processing**		
Detector	Falcon4	K3
Magnification	96,000	81,000
Voltage (kV)	300	300
Electron exposure ($e^-/Å^2$)	50	50
Defocus range (μm)	-1.0~-3.0	-1.0~-3.0
Pixel size (Å)	0.8	1.04
Symmetry imposed	C1	C1
Initial particle projections (no.)	2,300,698	4,199,012
Final particle projections (no.)	840,466	194,666
Map resolution (Å)	2.87	3.20
Map resolution range (Å)	2.80-3.50	3.00-4.50
FSC threshold	0.143	0.143
**Model Refinement**		
Refinement package	PHENIX-1.17.1-3660	PHENIX-1.17.1-3660
Real or reciprocal space	Real space	Real space
Model-Map CC (mask)	0.78	0.76
Model resolution (Å)	3.19	3.42
FSC threshold	0.5	0.5
B factors ($Å^2$, min/max/mean value)		
Protein residues	27.26/150.14/59.65	30.00/174.26/102.22
Ligands	58.10/58.10/58.10	66.32/72.29/69.19
**Model composition**		
Non-hydrogen atoms	8,081	8,170
Protein residues	1,028	1,041
R.m.s. deviations		
Bond lengths (Å)	0.003	0.004
Bond angles (°)	0.874	0.929
**Validation**		
MolProbity score	1.39	1.51
Clashscore	7.03	6.71
Rotamer outliers (%)	0.00	0.57
Ramachandran plot		
Favored (%)	98.51	97.27
Allowed (%)	1.49	2.73
Disallowed (%)	0.00	0.00
**Data availability**		
EMDB entry	EMD-34676	EMD-34677
PDB entry	8HDO	8HDP

Table S2. Effects of $A_{2B}R$ and its mutants on ADO- and BAY 60-6583-induced cAMP accumulations.

	cAMP accumulation assay (EC_{50}, μM)[a]		Cell-surface expression (Relative to WT)[a]
	Adenosine	BAY 60-6583	
WT	4.00±0.23	0.0422±0.00066	100±1.0
Empty vector	33.8±1.7	2.04±0.23	0
$Y10^{1.35}A$	33.6±1.2	3.17±0.86	57.6±0.69***
$A64^{2.61}I$	38.2±0.50***	2.71±0.43*	66.6±1.0***
$S68^{2.65}A$	3.80±0.06	0.0539±0.0076	94.3±1.0**
$V85^{3.32}A$	21.4±5.2	0.612±0.10*	98.0±0.99*
$T89^{3.36}A$	33.2±2.5**	1.74±0.45	107±0.9**
$I93^{3.40}A$	41.5±0.4***	4.36±0.81*	103±0.41*
$F173^{45.52}A$	41.5±6.2*	2.726±0.24**	89.1±1.5*
$E174^{ECL2}A$	4.20±0.17	0.0260±0.0019*	114±1.5**
$M179^{5.35}A$	16.8±2.1*	0.136±0.0052**	78.9±0.34**
$M182^{5.38}A$	18.7±1.1**	2.40±0.21**	97.5±1.2
$N186^{5.42}A$	21.0±2.7*	0.0431±0.0064	65.2±0.89***
$V191^{5.47}A$	41.8±5.5*	2.43±0.14**	74.7±1.4***
$W247^{6.48}A$	31.6±5.6*	0.926±0.039**	106±0.1*
$V250^{6.51}A$	40.9±2.8**	0.169±0.045	93.4±1.3
$V250^{6.51}L$	3.79±0.19	4.47±0.41**	119±1.1*
$H251^{6.52}A$	41.3±1.5**	4.22±0.37**	66.3±0.61***
$N254^{6.55}A$	42.1±0.39***	3.14±0.22**	54.2±0.41***
$M272^{7.35}A$	19.5±4.6	0.215±0.0056**	93.0±0.95
$I276^{7.39}A$	38.1±0.99***	2.96±0.15**	93.2±1.1*
$S279^{7.42}A$	45.1±2.5**	0.627±0.13*	97.6±1.4*
$H280^{7.43}A$	42.6±1.2***	4.18±0.55*	63.8±1.5**

[a] Data shown are means ± S.E.M. from at least three independent experiments. *P<0.01; **P<0.001 and ***P<0.0001 by one-way ANOVA followed by multiple comparisons test, compared with WT.

Table S3. Cell surface expression of $A_1R/A_{2A}R$ and its relative mutant at position 6.51 on BAY 60-6583-induced NanoBiT assay.

Receptor	Cell-surface expression (Relative to its WT)[a]
A_1R	100±0.94
A_1R-$L^{6.51}V$	94.5±0.38
$A_{2A}R$	100±0.62
$A_{2A}R$-$L^{6.51}V$	100±1.5

[a] Data shown are means ± S.E.M. from three independent experiments.

References

1 Duan J. et al. *Nat Commun* **11**, 4121 (2020).
2 Zhuang Y. et al. *Cell* **184**, 931-942 e918 (2021).
3 Rasmussen S.G. et al. *Nature* **469**, 175-180 (2011).
4 Guo H. et al. *IUCrJ* **7**, 860-869 (2020).
5 Rohou A. et al. *J Struct Biol* **192**, 216-221 (2015).
6 Zheng S.Q. et al. *Nat Methods* **14**, 331-332 (2017).
7 Zivanov J. et al. *Elife* **7**, (2018).
8 Punjani A. et al. *Nat Methods* **14**, 290-296 (2017).
9 Tunyasuvunakool K. et al. *Nature* **596**, 590-596 (2021).
10 Pettersen E.F. et al. *J Comput Chem* **25**, 1605-1612 (2004).
11 Emsley P. et al. *Acta Crystallogr D Biol Crystallogr* **60**, 2126-2132 (2004).
12 Adams P.D. et al. *J Synchrotron Radiat* **11**, 53-55 (2004).
13 Croll T.I. *Acta Crystallogr D Struct Biol* **74**, 519-530 (2018).
14 Pettersen E.F. et al. *Protein Sci* **30**, 70-82 (2021).
15 Inoue A. et al. *Cell* **177**, 1933-1947 e1925 (2019).
16 Draper-Joyce C.J. et al. *Nature* **597**, 571-576 (2021).
17 Lebon G. et al. *Nature* **474**, 521-525 (2011).
18 Jumper J. et al. *Nature* **596**, 583-589 (2021).
19 Jo S. et al. *J Comput Chem* **38**, 1114-1124 (2017).
20 Lee J. et al. *J Chem Phys* **153**, 035103 (2020).
21 He X. et al. *J Chem Phys* **153**, 114502 (2020).
22 Roe D.R. et al. *J Chem Theory Comput* **9**, 3084-3095 (2013).
